# Optimization and high‐density array of stereoelectroencephalography‐guided radiofrequency thermocoagulation for the treatment of pediatric tuberous sclerosis complex with epilepsy

**DOI:** 10.1111/cns.13804

**Published:** 2022-01-15

**Authors:** Tian Luo, Xinhua Wang, Ji Wang, Rui Zhao, Hao Li, Yuanfeng Zhou, Yi Wang

**Affiliations:** ^1^ Department of Neurology National Children’s Medical Center Children’s Hospital of Fudan University Shanghai China; ^2^ Department of Neurosurgery National Children’s Medical Center Children’s Hospital of Fudan University Shanghai China

**Keywords:** pediatric, radiofrequency thermocoagulation (RF‐TC), stereoelectroencephalography (SEEG), tuberous sclerosis complex

## Abstract

**Background:**

Tuberous sclerosis complex (TSC) is an autosomal dominant neurocutaneous syndrome involved in many organ systems. At the same time, epilepsy is the most common manifestation and more than 50% of TSC patients present with intractable epilepsy. This study investigated the efficacy and safety of optimized and high‐density stereoelectroencephalography (SEEG) guided radiofrequency thermocoagulation (RF‐TC) in treating TSC‐related epilepsy.

**Methods:**

Nine TSC children with refractory epilepsy were treated with first‐stage SEEG‐Guided RF‐TC, and four underwent second‐stage‐optimized high‐density array of SEEG‐Guided RF‐TC. Patients’ clinical data and postoperative outcomes were analyzed retrospectively.

**Results:**

The patients’ median age at surgery was 4 years and 2 month (range from 3 years and 5 month to 16 years and 7 month). The mean age at surgery was 6.7 years old. Eight in 9 (88.9%) patients achieved complete remission after the final operation at half‐year follow‐up. Of seven patients with final postoperative time beyond 1 year, 6 (85.7%) reached completely seizure‐free. No severe or long‐term neurologic impairment existed in all nine patients.

**Conclusion:**

Optimized high‐density array of SEEG‐guided RF‐TC is a safe and highly effective approach and can be an alternative application applied for TSC patients with refractory epilepsy.

## Graphical abstract

Optimized SEEG‐guided RF‐TC procedure was applied in nine TSC‐related refractory epilepsy children. 88.9% (8/9) patients acquired full remission at half‐year follow‐up after the final operation. 85.7% (6/7) patients with final postoperative time beyond 1 year reached completely seizure‐free. No serious or long‐term neurological impairment existed in all nine patients. Optimized high‐density array of SEEG‐guided RF‐TC is a safe and highly effective approach for TSC‐related refractory epilepsy.

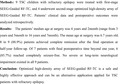

## 1 INTRODUCTION

Tuberous sclerosis complex (TSC) is an autosomal dominant neurocutaneous syndrome with 1 in 5000–10,000 live births.^1^, ^2^ Approximately 85% of TSC patients is associated with TSC1 (OMIM #191100) and TSC2 (OMIM #613254) gene mutations.^2^, ^3^ TSC can affect many different organ systems. Among the neurologic spectrum of seizure, intellectual disability, autism, and behavioral disorders, epilepsy is the most common manifestation, present in 80%–90% of TSC patients.^4^, ^5^ Despite standard yet compliant administration of anti‐seizure medicine (ASM), more than 50% of TSC patients still have pharmacy‐resistant epilepsy.^6^ Vagus nerve stimulation and the ketogenic diet have also been applied in TSC seizures with relatively good efficacy.^7^, ^8^ At the same time, epilepsy surgery is another alternative radical therapeutic approach for TSC patients considered potential candidates.^9^ Large‐size multicenter studies performed on respective epilepsy surgery of TSC showed that the rate of postoperative seizure‐free at 1‐year follow‐up reached 71%.^2^

The SEEG‐guided radiofrequency thermocoagulation (RF‐TC) technique was first reported in a feasibility study in 2004. The initial intention was to alleviate seizures by generating disorganization in the epileptogenic zone using a radiofrequency generator connected to the electrode contacts.^10^ This minimally invasive approach has been considerably applied in recent years due to its ability to perform well‐circumscribed lesion ablation, as well as stimulation and recording simultaneously.^10^, ^11^, ^12^ Up to now, SEEG‐guided RF‐TC has been indicated for patients with small, deeply seeded and well‐demarcated epileptogenic lesions such as periventricular nodular heterotopias,^13^ hypothalamic hematomas,^14^ and malformations of cortical development.^15^ However, there are very few relative articles published reporting the application of SEEG‐guided RF‐TC in the treatment of TSC with epilepsy. We utilized our optimized SEEG‐guided RF‐TC procedure to treat pediatric TSC with refractory epilepsy and achieved good clinical outcomes. To date, this report represents the first and most successful extensive case series of pediatric TSC epilepsy treated with SEEG‐guided RF‐TC procedure.

## 2 MATERIAL AND METHODS

### 2.1 Patients

Nine patients with a confirmed TSC and refractory epilepsy diagnosis (by the criteria of the International League Against Epilepsy) who underwent SEEG‐guided RF‐TC from May 1, 2018 to January 31, 2021, at Children's Hospital of Fudan University (Shanghai, China) were enrolled (Table 1). The study was performed following the ethical standards of the Declaration of Helsinki. It was within the Children's Hospital of Fudan University Ethics Board (No. 2016 [117]). Written informed voluntary consent was obtained from the parents of the child.

**TABLE 1:** General characteristics of the patients

Case no.	Gender	Mean age at seizure onset	Age at RF‐TC	Seizure frequency before RF‐TC	Electrodes no.	No. of nodular	Sizes of pathogenic nodule (cm)	Seizure type	SOZ	Complications during SEEG or RF‐TC	Times of RF‐TC	No. of coagulation sites	Developmental improvement	Seizure freedom at last visit	Seizure reduction%	Engel class	Follow up
1	F	3m	4y11m	1‐2/d	4	4	1.96*1.92*1.40	FS	R. precentral gyrus	/	1	2	+++	+	100	Ⅰ	3y3m
2	M	8m	4y2m	2/d	15	25	3.95*2.38*2.65	FS; FBTC	R. central operculum	Edema	1	1	+++	+	100	Ⅰ	2y9m
3	M	3y	13y	2‐4/d	11;6	21	2.20*1.25*1.45	FS	R. superior frontal gyrus	Fever	2 (13m interval)	7;3	+	+	100	Ⅰ	1y6m
4	M	4m	7y3m	1‐2/d	13	11	1.80*1.80*2.15	ES; FS	L. cuneus	Fever	1	13	+	−	100 (1 y)	Ⅲ	1y7m
5	F	8m	3y5m	1‐2/d	13;6	23	2.30*2.10*1.60	ES; GS	L. superior temporal gyrus	/	2 (10d interval)	1;1	+++	+	100	Ⅰ	1y4m
6	F	6m	16y7m	1‐2/d	8;6	5	1.23*1.40*1.27	FS; FBTC	L. post insular sulcus	/	2 (6m interval)	1;1	−	−	85	Ⅲ	6m
7	F	9m	3y1m	1‐8/d	7	3	2.50*1.43*1.90	ES; FS	L. orbital gyrus	/	1	1	+++	+	100	Ⅰ	12m
8	M	1d	3y5m	3‐5/d	12;5	14	1.50*1.20*2.20	ES; FS	R. paracentral lobule	Fever; transient pain loss and lameness	2 (15d interval)	1;1	+++	+	100	Ⅰ	12m
9	M	3y5m	4y2m	3‐7/d	8	2	2.00*2.10*2.20	FS	L. frontal pole	/	1	1	+++	+	100	Ⅰ	9m

Abbreviations: ES, epileptic spasm; FBTC, focal to bilateral tonic clonic; FS, focal seizure; GS, gelastic seizure; L, left; No, number; R, right; SOZ, seizure‐onset zone.

### 2.2 Presurgical examination

All patients underwent a comprehensive noninvasive phase Ⅰ presurgical examination, including long‐term scalp video‐EEG monitoring, high‐resolution MRI (3Tesla), 18F‐fluorodeoxyglucose‐positron emission tomography (FDG‐PET) and neuropsychological testing. The neuroimaging of MRI requires a three‐dimensional (3D) T1‐weighted sequence (1 × 1 × 1 mm^3^, no‐contrast and double‐contrast sequences are included, the latter can display blood vessels), axial, coronal and sagittal T2 fluid‐attenuated inversion recovery (FLAIR) sequence (3 mm slice thickness). Opportunities and strategies for electrodes implantation were decided following a systematic and multidisciplinary presurgical evaluation upon the information obtained above.

### 2.3 First‐stage SEEG implantation to locate pathogenic nodule and optimize RF‐TC

**FIGURE 1:**
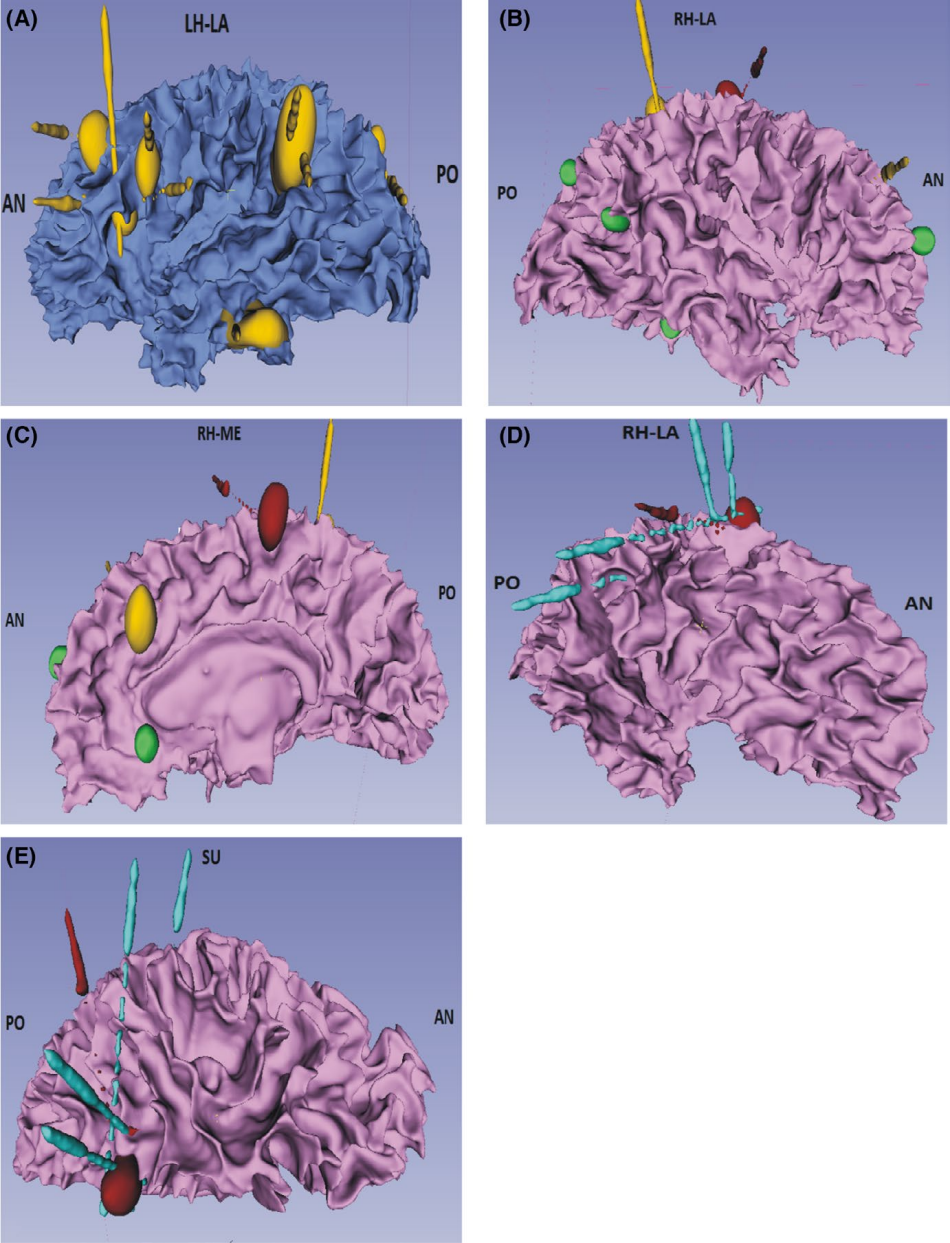
Schematic protocol of first (A, B, and C) and second‐stage(D and E)‐optimized and high‐density SEEG implantation prepared for RF‐TC procedure in patient No. 8. Fourteen nodules and 12 electrodes were applied in the first stage of SEEG implantation. All the nodules and electrodes were reconstructed in the 3D imaging of white matter. Red nodule: pathogenic nodule confirmed by SEEG, Yellow nodules: non‐pathogenic nodules confirmed by SEEG, Green nodule: nodules without implanted electrodes. (A) Lateral view of left hemisphere: six suspicious nodules were implanted with nine electrodes, and three were both crossed with two electrodes. (B, C) Lateral and medial view of the right hemisphere: three suspicious nodules were implanted with three electrodes, and a red nodule located at the paracentral lobule was the pathogenic nodule. (D, E) Lateral and superior view of the right hemisphere: four additional blue electrodes were implanted, and high dense three‐dimensional cross‐bonding electrodes were planned with an original red electrode. AN, anterior; PO, posterior; LA, lateral; ME, medial; SU, superior; LH, left hemisphere; RH, right hemisphere

All the TSC patients with epilepsy underwent SEEG implantation following the modified protocol^16^ and individualized anatomic‐electro‐clinical hypothesis. The principle is to locate the pathogenic nodule and optimize the possibility of maximal coagulation sites.^17^ A frameless robot‐assisted SEEG system (Sino‐precision) performs the implantation procedure with patients under general anesthesia conditions in the operating room. Reconstruction of subject‐specific SEEG electrodes localization was performed based on the combination of pre‐and post‐implantation images (Figure 1A–C).

**FIGURE 2:**
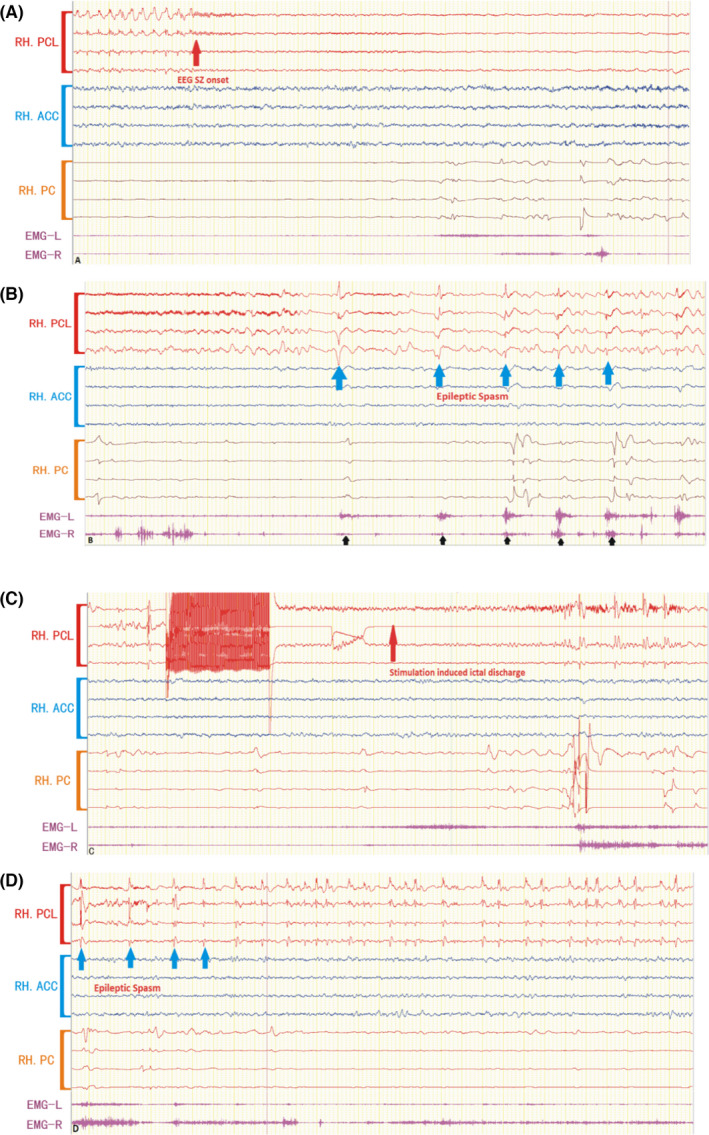
Stimulation‐induced seizures during electrodes implantation in patient No. 8. The ictal discharge of habitual seizure started at the RH.PCL. (A, B) Ictal discharges originated from the R.PCL with very low voltage fast activity (red arrow) and then spike rhythm recruitment followed by a repetitive burst of polyspike‐slow wave (blue arrow), accompanied by epileptic spasm characterized by a phasic contraction, polymorphically with rhombus appearance (black arrow) was observed. (C, D) electrical stimulation‐induced ictal discharge (red arrow) and habitual seizure (blue arrow) at RH.PCL. RH, right hemisphere; PCL, paracentral lobule; ACC, anterior cingulate cortex; PC, Parietal cortex; EMG‐L, Electromyogram of Left deltoid muscle; EMG‐R, Electromyogram of Right deltoid muscle

After implantation, the invasive SEEG exploration for pathogenic nodule was performed. Functional mappings are also assessed during the SEEG recording. A further systematic evaluation on the eligible patients for the SEEG‐guided RF‐TC was scheduled according to the localization of the pathogenic nodule and the functional mapping data (Figure 2A–D **)**. An eligible SEEG recording site for RF‐TC meets one or more of the following criteria: (1) showed spike‐wave discharges or seizure‐onset pattern of low amplitude fast electrical activities; (2) intralesional location; (3) induction of habitual ictal discharge by tapering drugs or electrical stimulation.^11^, ^15^ Reasons for being excluded from RF‐TC may include the following: pathogenic nodule involved in the critical functional area; the procedure may create new unacceptable neuropsychological deficits; no definite pathogenic nodule identification; the thermocoagulation electrode site is too close to the main blood vessel; and patient's refusal.^15^

### 2.4 Second‐stage‐optimized high‐density array of SEEG‐guided RF‐TC

The second‐stage high‐density SEEG‐guided RF‐TC was recommended to patients when they had residual seizures or seizure recurrence after the first coagulation procedure of pathogenic nodules. According to the size, shape and location of pathogenic nodule identified at the first stage, high‐dense three‐dimensional cross‐bonding electrodes were designed (the trajectories were planned both orthogonally and obliquely, and the orthogonal and oblique electrodes implanted in different directions formed a three‐dimensional array) to provide an expanded scope of ablation area. (Figure 1D,E).

### 2.5 SEEG‐guided RF‐TC scheme

We performed bipolar coagulation by gradually increasing the power from 0 to 3.5 W over 15 s, holding the coagulation power at 3.5 W for 45 s, which caused a local temperature up to 78–82℃ and a lesion of 5–7 mm in diameter and 3.5 mm in thickness between two connected or adjacent contacts of one electrode, two electrodes or more cross‐bonding electrodes.^14^, ^15^, ^18^, ^19^ The number of coagulation sites is variable according to each patient's situation. Patients were under real‐time monitoring and clinical evaluation during the whole procedure, then the electrodes were removed. The electrodes (Alcis) had 5 to 18 contacts, with each contact 2 mm in length and 0.8 mm in diameter (intercontact spacing 1.5 mm). Lesions were made using a radiofrequency lesion generator system (COSMAN RFG‐1A).

### 2.6 Follow‐up

**FIGURE 3:**
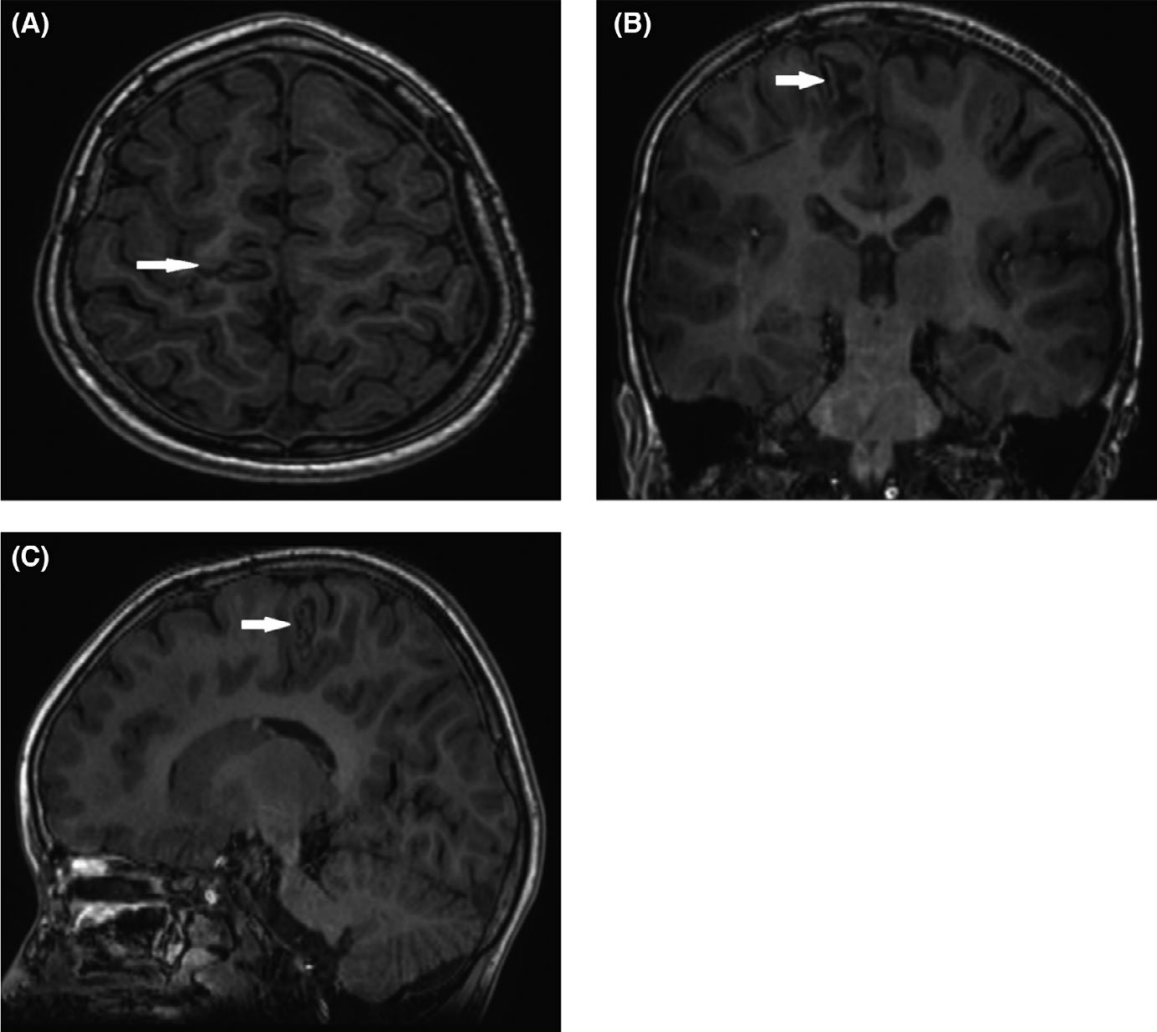
Postoperative MR images were obtained 6 months after the second stage of high‐density RF‐TC in patient No. 8. The expanding coagulated lesion (white arrow) at the nodule of the right paracentral lobule was shown (A, axial image; B, coronal; C, sagittal)

According to Engel's classification system, the post‐RF‐TC outcome was usually evaluated at 1, 3, 6, and 12 months, including the seizure outcomes. Routine MRI (Figure 3A–C) was also performed to confirm the thermocoagulation lesion.

## 3 RESULTS

### 3.1 Demographics

A summary of the general characteristics and critical points of RF‐TC in each patient is presented in Table 1. None of them had previously undergone open surgery. The patients’ median age at surgery was at the age of 4 years and 2 month (range from 3 years and 5 month to 16 years and 7 month), and the median age at seizure onset was at the period of 8 months (range from 1 day to 3 years and 5 months). All nine patients had TSC‐associated drug‐resistant epilepsy, and seizure types include focal seizure, epileptic spasm, gelastic seizure, and focal to a bilateral tonic‐clonic seizure. The median number of implanted electrodes at first‐stage was 11 (range from 4 to 15), and the median number of implanted electrodes at second stage was 6 (range from 5 to 6). All nine patients suffered mild‐to‐severe developmental delay before the RF‐TC procedure.

### 3.2 SEEG findings

Interictal spikes, spontaneous, and induced seizures, which may reflect the location of pathogenic and epileptogenic tubers were recorded in all nine patients. The habitual or automatic seizures were observed during the most time. Electrical stimulation of the nodular could reproduce seizures in all nine patients. All patients can record ictal discharge near the interface and in a deeper area of the epileptogenic nodules.

### 3.3 Seizure outcomes

**FIGURE 4:**
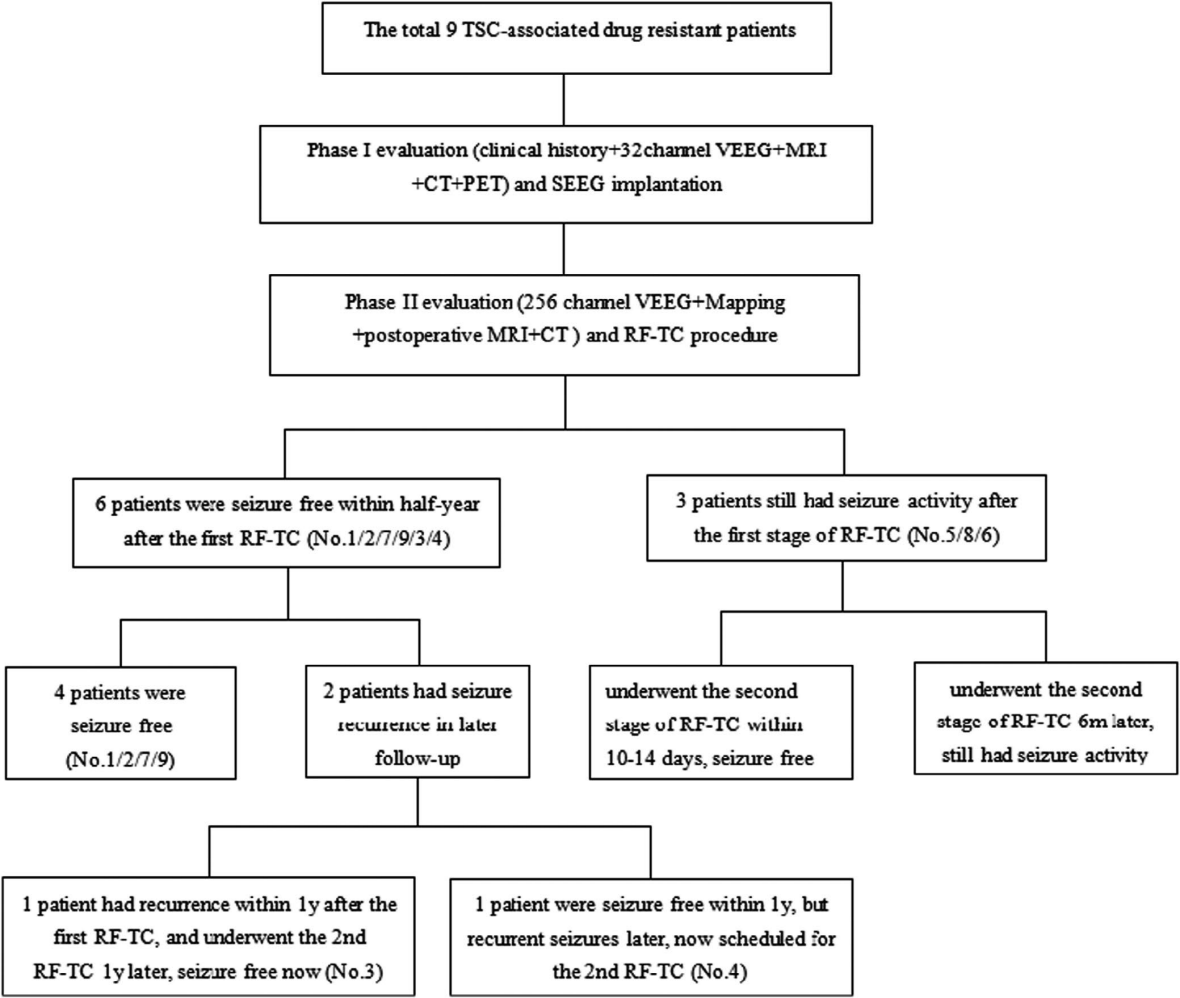
Overlook procedure and outcome of RF‐TC treatment in our 9 pediatric TSC

The last follow‐up was delivered on August 18, 2021. The follow‐up duration ranges from 6 to 39 months, with a median of 16 months. The reduction of seizure frequency was compared with the pre‐RF‐TC periods. All patients were responders (R+, seizure frequency reduction of at least 50%) to RF‐TC. Among the nine patients, 7 (77.8%) were seizure‐free now. Four patients kept completely seizure‐free up till now (No. 1/2/7/9). Patient No. 3 experienced recurrence within 1 year after the first RF‐TC and underwent an additional high‐density RF‐TC procedure 1 year later. The seizure has been completely controlled for more than 18 months now. Patient No. 4 was kept seizure‐free for more than 1 year but underwent a recurrence from the 13th month after the RF‐TC procedure. The seizure type changed from epileptic spasm to transient tonic seizure, now preparing for a second pre‐surgery evaluation. Three patients still had seizures after the first RF‐TC, two patients scheduled a short‐term second RF‐TC, and were seizure‐free for more than 1 year now (No. 5/8). Patient No. 6 underwent the second RF‐TC 6 months later, and she had seizure activity now. Still, the seizure frequency had an apparent reduction from 1–2 times/day to 1 time/week with shorter seizure duration from average 3 to 1 min (Figure 4).

### 3.4 Complications

Three of nine patients underwent a low fever, which recovered within 3–5 days after the procedure. Infrequently, a post‐surgery lameness was recorded in patient No. 8 and restored to normal within 2 months. Routine mannitol and dexamethasone were administrated to alleviate the local brain swelling around the coagulated region. Other complications, including infection, bleeding, or other permanent neurologic deficits, were not found in all nine patients.

## 4 DISCUSSION

TSC is a genetic disorder with various clinical features, predominantly developing benign tumors (hamartomas) in multiple organs. Epileptogenic tubers can be genesis in the central nervous system of cortical and subcortical locations.^20^, ^21^ Despite the numerous available ASM applied, refractory epilepsy still counts for a large proportion in TSC patients.^22^ In recent years, alternative non‐pharmaceutical therapies have been developed for managing refractory epilepsy in TSC patients to relieve or remove the original seizure activity from the responsible lesion. Although resection surgery has been applied in TSC epilepsy patients and achieved good efficacy nowadays, the exploration of minimally invasive but equal or even better efficacy approaches has been an interest for neurosurgery experts.

After a detailed and cogitative evaluation of the indication and contraindication, we applied SEEG‐guided RF‐TC in nine TSC‐related refractory epilepsy patients and obtained superior efficacy. After the first procedure of RF‐TC, three patients still had seizure activity. As for patients No. 5&8, additional RF‐TC procedures were scheduled within 2 weeks. As the primary shortage of SEEG‐guided RF‐TC is the limited extent of ablation areas,^23^ when the second RF‐TC proceeded, we chose a high dense 3D cross‐bonding contacts array among various electrodes to maximally destroy the epileptogenic nodule confirmed at first‐stage SEEG‐guided RF‐TC.

Considering two patients underwent a second operation half or 1 year later, two kinds of follow‐up dates were calculated to reflect the efficiency of the RF‐TC procedure accurately. That's the follow‐up date apart from the first and the final RF‐TC operation. The completely seizure‐free proportion reached 66.7% (6/9) half‐year after the first operation. Seven patients achieved postoperative complete seizure freedom of 62.5% (5/8) at a 1‐year follow‐up. When the follow‐up date is calculated based on the final RF‐TC procedure, 88.9% (8/9) acquired complete remission after the operative at half‐year follow‐up; Of 7 patients with postoperative time beyond 1 year, 6 (85.7%) reached completely seizure‐free, which indicates some patients can also achieve a complete remission by an additional RF‐TC procedure if the first RF‐TC was not entirely valid. When calculated by the final RF‐TC, our results were more effective than the remission rate of large‐scale TSC resection surgery patients, which achieved a seizure freedom percentage of 71% (258/364) at 1 year postoperative follow‐up.^2^

The multivariate analyses of TSC resection operation concluded that the surgical success rate is mainly related to the total resection of epileptogenic tubers, rather than some other factors such as seizure type, the number of tubers, surgical approach, or age at seizure onset.^2^ Similar conclusions can be drawn from our sample of RF‐TC procedure details and results. From the seizure outcomes, we find that the procedure efficacy is more significantly associated with the anatomic location and size of the epileptogenic nodules compared with other factors as seizure type, the number and size of nodules, or even the number of coagulation sites.

No severe or long‐term neurologic impairment exists in the nine patients except for the transient fever, edema or pain loss, which further verify the security of the RF‐TC procedure.^24^ By contrast, resection operation may result in intracranial hematoma and permanent complications of hemiplegia.^2^

Although there had been a sizeable systematic review of SEEG‐guided RF‐TC in focal epilepsy patients, very few applications of RF‐TC had been reported in tuberous sclerosis. This paper introduces successful cases of small‐scale SEEG‐guided RF‐TC applications in TSC epilepsy treatment. Besides the minimally invasive RF‐TC operation, some other less invasive approach exists, such as laser interstitial thermal therapy procedure, which is mainly suitable for definite pathogenic lesions and not for multiple lesions, such as TSC. As it is operated under the MRI guidance, real‐time monitoring of intraoperative ictal discharge conditions cannot be performed.^25^

By contrast, the SEEG‐guided RF‐TC is suitable for multiple lesions and real‐time monitoring epileptic focus as well as ablation of the epileptogenic zone simultaneously. For patients with recurrence, a second high‐density three‐dimensional cross‐bonding of RF‐TC procedure can also expand the ablation area and provide the final epilepsy remission, which can be considered an alternative treatment for TSC‐related refractory epilepsy in future.

## 5 CONCLUSION

SEEG‐guided RF‐TC is a highly effective and safe approach applied for TSC patients with refractory epilepsy. Although the first stage of SEEG exploration and the ablation volume may be restricted due to the number of electrodes, the second stage of stereotactic high‐density 3D cross‐bonding RF‐TC enables maximum ablation. It can also achieve final seizure remission. SEEG‐guided RF‐TC can be an alternative application for the treatment of TSC with drug‐resistant epilepsy.

## CONFLICT OF INTEREST

All the authors declare that they have no known competing financial interests or personal relationships that could influence the work reported in this paper.

## AUTHORS’ CONTRIBUTIONS

TL is the first author who analysis the data and drafted the manuscript. XHW and JW conceived the idea and supervised the study. RZ and HL major participated in radiofrequency thermocoagulation procedures. YFZ and YW are the corresponding authors who revised and were responsible for the manuscript content. All authors participated in the proofreading. All the authors have read and approved the final manuscript.

## Data Availability

The datasets used during or analyzed during the current study are available from the corresponding author on request.
